# Excessive daytime sleepiness is associated with relative delta frequency power among patients with mild OSA

**DOI:** 10.3389/fneur.2024.1367860

**Published:** 2024-04-05

**Authors:** Timothy Howarth, Masoumeh Tashakori, Tuomas Karhu, Matias Rusanen, Henna Pitkänen, Arie Oksenberg, Sami Nikkonen

**Affiliations:** ^1^Department of Technical Physics, University of Eastern Finland, Kuopio, Finland; ^2^Darwin Respiratory and Sleep Health, Darwin Private Hospital, Darwin, NT, Australia; ^3^College of Health and Human Sciences, Charles Darwin University, Darwin, NT, Australia; ^4^Diagnostic Imaging Center, Kuopio University Hospital, Kuopio, Finland; ^5^HP2 Laboratory, INSERM U1300, Grenoble Alpes University, Grenoble Alpes University Hospital, Grenoble, France; ^6^Sleep Disorders Unit, Loewenstein Hospital – Rehabilitation Center, Ra’anana, Israel

**Keywords:** excessive daytime sleepiness, MSLT, OSA, ESS, power spectral densities

## Abstract

**Background:**

Excessive daytime sleepiness (EDS) is a cause of low quality of life among obstructive sleep apnoea (OSA) patients. Current methods of assessing and predicting EDS are limited due to time constraints or differences in subjective experience and scoring. Electroencephalogram (EEG) power spectral densities (PSDs) have shown differences between OSA and non-OSA patients, and fatigued and non-fatigued patients. Therefore, polysomnographic EEG PSDs may be useful to assess the extent of EDS among patients with OSA.

**Methods:**

Patients presenting to Israel Loewenstein hospital reporting daytime sleepiness who recorded mild OSA on polysomnography and undertook a multiple sleep latency test. Alpha, beta, and delta relative powers were assessed between patients categorized as non-sleepy (mean sleep latency (MSL) ≥10 min) and sleepy (MSL <10 min).

**Results:**

139 patients (74% male) were included for analysis. 73 (53%) were categorized as sleepy (median MSL 6.5 min). There were no significant differences in demographics or polysomnographic parameters between sleepy and non-sleepy groups. In multivariate analysis, increasing relative delta frequency power was associated with increased odds of sleepiness (OR 1.025 (95% CI 1.024–1.026)), while relative alpha and beta powers were associated with decreased odds. The effect size of delta PSD on sleepiness was significantly greater than that of either alpha or beta frequencies.

**Conclusion:**

Delta PSD during polysomnography is significantly associated with a greater degree of objective daytime sleepiness among patients with mild OSA. Further research is needed to corroborate our findings and identify the direction of potential causal correlation between delta PSD and EDS.

## Introduction

Excessive daytime sleepiness (EDS) is highly prevalent across populations, estimated to affect up to one in five people (1). EDS is associated with an increased risk of motor vehicle accidents (2, 3), decreased quality of life (4, 5), reduced work productivity (4, 6), and significant economic burden (7, 8). In addition, EDS has shown significant associations with psychological distress (9, 10), depression and bipolar disorder (11–15), and seasonal affective disorder (11). EDS is often associated with sleep disorders, such as insomnia, narcolepsy, or obstructive sleep apnoea (OSA). Among patients with OSA, a greater severity as judged by the apnoea-hypopnoea index (AHI) has been associated with EDS (16). Yet, alongside increasing OSA severity there appears to be a concurrent increase in comorbidities (17, 18), which are also associated with increased daytime sleepiness (19–22), and may thus confound the effect of OSA on EDS. However, EDS is common even among patients with only mild OSA (AHI 5–15), without concomitant insomnia or narcolepsy (16), and a significant proportion of patients who are receiving continuous positive airway pressure (CPAP) therapy still suffer from EDS (23–25). The current prevailing theory suggests that EDS in the context of OSA is caused by a combination of sleep fragmentation and intermittent hypoxia, which over time lead to neuronal damage (26). Yet, although these markers are less common among patients with mild OSA, these patients are still prone to EDS, and therefore further exploration into the potential underlying mechanisms is warranted. Furthermore, studies which have utilised subjective markers of sleepiness have found contradictory results in polysomnographic parameters between sleepy and non-sleepy patients (27, 28).

Alternate parameters assessed through polysomnography (PSG) are being increasingly investigated for their relationship with EDS. Oxygen desaturation severity and power spectral densities (PSDs) for example have shown greater correlations with EDS as measured via multiple sleep latency tests (MSLTs) mean sleep latency (MSL) than either the AHI or the oxygen desaturation index (ODI) (29, 30). Although power spectral analyses are commonly used to quantify electroencephalogram (EEG) outcomes, the association of these with EDS is sparsely reported, though associations with other somnolence or psychiatric disorders have been (31–34). Within the sleep field, EEG-based PSDs are typically analysed at four frequency bands – delta (δ) (0.5–4 Hz), theta (θ) (4–8 Hz), alpha (α) (8–12 Hz), and beta (β) (12–30 Hz), though the exact thresholds used between studies may differ. Among patients with insomnia, beta frequency power has been found to be higher in non-rapid eye movement (NREM) stages, but lower in rapid eye movement (REM) stages compared to patients without insomnia (35). Among patients with narcolepsy, alpha power has been found to be higher in REM stages and delta power lower in NREM stage 1 (N1) compared to controls (36). Concerning EDS, one recent study reported increased delta and reduced alpha and beta power prior to sleep among patients classified as sleepy [Epworth sleepiness scale (ESS) score > 20] compared to non-sleepy patients (ESS score < 5) (37). Another reported greater alpha power among drowsy compared to non-drowsy patients (also assessed via ESS) – though in a resting but non-sleep state (38). To date, however, no studies have reported on associations between sleep EEG PSDs and objectively measured EDS via MSLTs which may have higher generalisability than subjective measures of EDS (39).

Therefore, this study aimed to describe the associations between objectively measured EDS and the EEG PSDs assessed over the whole night among patients with mild OSA. Based on previous findings in different patient populations, we hypothesised that during N3 and REM sleep, alpha PSD would be increased, and delta PSD decreased among suspected OSA patients with EDS compared to those without. Furthermore, we hypothesised there would be a significant positive correlation between delta PSD and MSL.

## Methods

### Dataset

Between the years 2001 and 2011, patients were referred to Loewenstein Hospital rehabilitation centre (Raanana Israel) for an overnight PSG [level 1 study, analysed with REMbrandt Manager System (Medcare CO, Amsterdam, Netherlands) and following day MSLT] [following AASM guidelines (40)] based on suspicion for OSA alongside complaints of daytime sleepiness. The PSG data were rescored for research purposes at Kuopio University Hospital according to the AASM 2007 guidelines and clinical practices at the time. The MSL was determined by calculating the mean of the four nap recordings in the MSLT.

Patient demographic and anthropometric information were collected by the sleep technologist prior to the PSG. From the initial cohort (*n* = 937), patients with missing demographic/clinical data (*n* = 104), absence of sleep stage scoring (*n* = 2), less than 6 h of total sleep time (*n* = 29), or failed MSLTs (*n* = 10) were excluded from the analysis. Furthermore, this study focused on patients with mild OSA (5 ≤ AHI <15 events/h), and thus 139 patients were included in the final analysis. Patients were categorized as ‘sleepy’ or ‘non-sleepy’ based on their MSL with the sleepy group including patients with an MSL <10 min.

### EEG processing

Six EEG recordings were conducted across the frontal, central, and occipital regions, and the placement of electrodes for these recordings followed the International 10–20 System guidelines (41). These signals were sampled at 256 Hz and imported to MATLAB 2021b (MathWorks Inc., Natick, Massachusetts, United States) for further analysis. The central EEG signals (C3-A2 and C4-A1), being more prevalent among the patients, were selected for comparison between the sleepy and non-sleepy groups. For this purpose, the EEG signals were filtered using a fifth-order Chebyshev Type I bandpass filter with 0.3 and 35 Hz cutoff frequencies. The filtered signals were divided into 30-s epochs according to sleep stages. The analysis included epochs identified as light sleep (N1 + N2), deep sleep (N3), and REM.

In frequency domain analyses, the PSD was estimated within each 30-s epoch by Welch’s method with 50% overlap and employing a Hamming window with size 1,000 points. The relative PSDs were calculated across various frequency bands, and for this study defined as slow oscillation (0.3–1 Hz), delta (1–4 Hz), theta (4–8 Hz), alpha (8–12 Hz), beta (12–30 Hz), and gamma (30–35 Hz). Relative PSDs were determined by dividing the PSD values for each specific frequency band (alpha, beta, and delta) by the total PSD calculated over the frequency range of 0.3 to 35 Hz. Then, the median relative PSDs were calculated for each signal in each sleep stage. Based on preliminary results, the frequency bands delta, alpha, and beta were selected for further analysis.

### Ethics approval

Data collection and processing were approved by the Ethical Committee of the Loewenstein Hospital – Rehabilitation Center (0006-17-LOE).

### Statistical analysis

The Mann–Whitney U test was used to test for statistically significant differences in continuous variables in demographics, polysomnography variables (total sleep time, night sleep latency, wakening after sleep onset (WASO), percentage time in NREM (N1 + N2 + N3) and REM stages, apnoea-hypopnea index, oxygen desaturation index (defined as a drop of ≥4%) and time under 90% oxygen saturation as a percentage of total sleep time (T90%)), and relative EEG frequency band powers between sleepy and non-sleepy groups. Furthermore, the Chi-squared test was employed to evaluate the statistical significance of categorical values.

To explore the predictive efficacy of EEG PSDs on EDS, six binomial logistic regression models were developed utilising relative PSD in each frequency band (alpha, beta and delta in both C3A2 and C4A1 channels) as the primary predictor. For the regression analyses, the relative band powers were scaled to the range of 0 to 100 by multiplying the values by 100 to make reported odds ratios (ORs) and 95% confidence intervals (CIs) more easily interpretable and comparable. The models were adjusted for age, sex, BMI, REM, and T90. Due to potential multiple comparison issues, a *p*-value threshold of 0.01 was considered for statistical significance. Post-hoc Wilcoxon tests were employed to compute the effect size and to allow comparisons of effect between frequency bands (alpha, beta, and delta) and between the C3A2 and C4A1 channels. The Wilcoxon effect size was calculated as the z-statistic dividing by the square root of the sample size.

## Results

Of the 139 patients with mild OSA included, 66 (47%) were categorised as non-sleepy (median MSL = 14 min) and 73 (53%) as sleepy (median MSL = 6.5 min). Patients in the sleepy and non-sleepy groups were predominantly males; however, there was a statistically significant difference in gender distribution between the groups (*p* = 0.007; Table 1). Sleep architecture, AHI and ODI did not significantly differ between sleepy and non-sleepy groups.

**Table 1:** Demographic data and sleep characteristics of sleepy and non-sleepy groups.

Demographic				
	All patients (*n* = 139)	Non-sleepy (*n* = 66)	Sleepy (*n* = 73)	*p*-value
Age (years)	51 (42–57)	51 (42–58)	50 (44–57)	0.86
Male n (%)	103 (74.1%)	**42 (63.6%)**	**61 (83.5%)**	**0.007**
BMI (Kg/m^2^)	28.9 (26.3–32.6)	28.9 (26.5–32.9)	29.1 (25.9–31.5)	0.71
Normal weight (18.5 ≤ BMI < 25)	19 (13.7%)	9 (13.6%)	10 (13.7%)	0.99
Overweight (25 ≤ BMI < 30)	59 (42.4%)	28 (42.4%)	31 (42.5%)	0.10
Obese (BMI ≥ 30)	61 (43.8%)	29 (43.9%)	32 (43.8%)	0.99
**Polysomnography**				
Total sleep time (min)	405 (390.6–420.2)	400.2 (391.0–419.5)	407 (390.4–421.1)	0.50
Night sleep latency (min)	8.1 (5.0–13.4)	9.4 (4.9–19.5)	7.5 (5.0–10.5)	0.02
WASO (min)	22 (11.6–43.8)	22.2 (11.0–46.5)	22.0 (12.8–38.2)	0.91
Wake (%)	8.2 (4.6–14.5)	9.4 (4.6–15.6)	8.0 (4.9–11.6)	0.45
N1 (%)	1.8 (0.4–3.5)	1.8 (0.2–3.5)	1.9 (0.5–3.5)	0.54
N2 (%)	48.1 (42.6–55.5)	48.4 (40.8–56.2)	48.0 (43.3–55.4)	0.83
N3 (%)	20.6 (15.4–26.4)	19.8 (14.5–26.6)	20.7 (17.0–25.8)	0.72
NREM (%)	72.0 (67.3–77.3)	72.1 (64.8–77.5)	71.9 (69.2–77.0)	0.95
REM (%)	18.1 (13.9–21.4)	17.7 (13.3–23.1)	18.3 (14.5–21.0)	0.32
AHI (events/h)	9.8 (7.4–12.9)	9.8 (7.3–12.9)	9.8 (7.6–12.6)	0.83
ODI (events/h)	6.8 (3.9–11.0)	6.6 (3.5–11.7)	6.9 (4.1–10.4)	0.80
T90 (%)	0.3 (0.1–1.9)	0.4 (0.1–1.9)	0.2 (0.1–1.6)	0.84
**MSLT**				
MSL (min)	9.5 (6.4–13.9)	**14.0 (11.8–16.6)**	**6.5 (5.1–8.1)**	**3e-24**

Statistical significance was calculated with the Mann–Whitney U test (for continuous variables) or the Chi-squared test (for categorical variables). Bolded values were considered significant (*p*-values<0.01). All parameters are presented as median (IQR). AHI, apnea-hypopnea index; BMI, body mass index, IQR, Interquartile range; MSL, mean sleep latency; MSLT, Multiple sleep latency test; NREM, non-rapid eye movement sleep; ODI, oxygen desaturation index; REM, rapid eye movement sleep; T90%, percent sleep time below 90% oxygen saturation; WASO, Wake after sleep onset.

**Figure 1:**
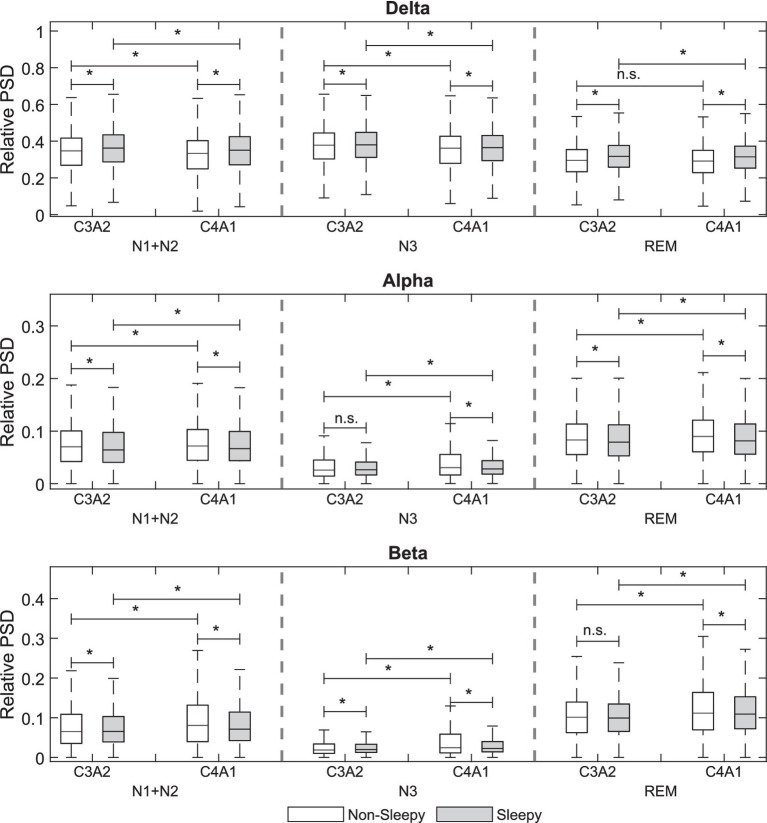
Comparison of the relative electroencephalogram power spectral densities (PSD) between sleepy and non-sleepy groups in N1 + N2, N3, and REM for C3A2 and C4A1 channels. *p* < 0.01 = *.

Significant differences in relative PSDs between sleepy and non-sleepy patients were noted in each sleep stage, in both C3A2 and C4A1 channels (Figure 1, values provided in Supplementary Table S1). During N1 + N2 stages sleepy patients showed significantly higher delta power, and reduced alpha and beta power, though the reduced alpha was only evident in the C3A2 channel. Among sleepy patients, the C3A2 channel showed significantly higher delta power, and lower alpha and beta power than the C4A1 channel. Among non-sleepy patients however, though the delta power in C3A2 was significantly higher than in the C4A1, the beta power was also significantly greater in C3A2, with no difference in alpha power between channels. Similar results were seen in N3 stage, with sleepy patients showing significantly increased delta, and reduced alpha and beta, though the reduced beta was this time seen only in the C4A1 channel. For both sleepy and non-sleepy patients, delta power was higher in C3A2 compared to C4A1, while alpha and beta powers were lower. In REM stage, sleepy patients had higher delta power, and lower alpha and beta power compared to non-sleepy patients. There was no significant difference in delta power between channels, however alpha and beta power were significantly reduced in the C3A2 channel.

In multivariate binomial regression (adjusted for age, sex, REM, BMI & T90%) relative PSDs at each frequency band showed significant associations with odds for EDS. Increased delta power PSD significantly increased odds of EDS while increased alpha and beta powers significantly decreased the odds for EDS (Tables 2, 3).

**Table 2:** Odds ratios (ORs) of being sleepy based on relative EEG band powers.

Channel	Predictors	Delta		Alpha		Beta	
		OR (95% CI)	*p*-value	OR (95% CI)	*p*-value	OR (95% CI)	*p*-value
C3A2	Age (unit - 1 year)	1.009 (1.008–1.010)	<0.001	1.007 (1.006–1.008)	<0.001	1.008 (1.007–1.009)	<0.001
	BMI (unit - 1 kg/m^2^)	1.035 (1.033–1.037)	<0.001	1.030 (1.029–1.032)	<0.001	1.030 (1.028–1.032)	<0.001
	Sex (male)	4.283 (4.142–4.429)	<0.001	4.034 (3.903–4.170)	<0.001	4.134 (3.998–4.274)	<0.001
	REM	1.108 (1.072–1.146)	<0.001	0.967(0.936–0.999)	0.049	1.036 (1.002–1.072)	0.037
	T90% (unit - %)	1.039 (1.037–1.041)	<0.001	1.036 (1.034–1.038)	<0.001	1.035 (1.033–1.037)	<0.001
	Corresponding PSD (unit – 1% in scaled range)	1.025 (1.024–1.026)	<0.001	1.001 (0.998–1.003)	0.594	0.983 (0.981–0.985)	<0.001
C4A1	Age (unit - 1 year)	1.008 (1.007–1.011)	<0.001	1.007 (1.006–1.008)	<0.001	1.008 (1.007–1.009)	<0.001
	BMI (unit - 1 kg/m^2^)	1.036 (1.034–1.038)	<0.001	1.032 (1.031–1.034)	<0.001	1.036 (1.033–1.038)	<0.001
	Sex (male)	4.438 (4.291–4.591)	<0.001	4.118 (3.984–4.257)	<0.001	4.481 (4.332–4.634)	<0.001
	REM	1.078 (1.043–1.114)	<0.001	0.987 (0.955–1.021)	0.460	1.093 (1.058–1.131)	<0.001
	T90% (unit - %)	1.041 (1.038–1.043)	<0.001	1.035 (1.033–1.037)	<0.001	1.035 (1.033–1.037)	<0.001
	Corresponding PSD (unit - 100)	1.027 (1.026–1.028)	<0.001	0.989 (0.986–0.992)	<0.001	0.970 (0.968–0.971)	<0.001

Binomial logistic regression analyses were adjusted for age, BMI, sex, REM, and T90%. BMI, body mass index; CI, Confidence interval; OR, odds ratio; PSD, Power spectral density; REM, Rapid eye movement; T90%, percentage of total sleep time under 90% oxygen saturation.

**Table 3:** Odds ratios (ORs) of being sleepy for univariate binomial regression.

Predictors		OR (95% CI)	*p*-value
Age (unit - 1 year)		1.002 (1.001–1.004)	<0.001
BMI (unit - 1 kg/m^2^)		1.015 (1.013–1.016)	<0.001
Sex (male)		2.977 (2.890–3.067)	<0.001
REM		0.960 (0.931–0.991)	0.011
T90% (unit - %)		1.028 (1.026–1.029)	<0.001
Corresponding PSD (unit – 1% in scaled range)	Delta_C3A2	1.016 (1.015–1.017)	<0.001
	Alpha_C3A2	0.994 (0.992–0.996)	<0.001
	Beta_C3A2	0.991 (0.988–0.993)	<0.001
	Delta_C4A1	1.017 (1.016–1.018)	<0.001
	Alpha_C4A1	0.989 (0.987–0.992)	<0.001
	Beta_C4A1	0.979 (0.978–0.981)	<0.001

BMI, body mass index; CI, Confidence interval; OR, odd ratios; PSD, Power spectral density; REM, Rapid eye movement; T90%, percent sleep time below 90% oxygen saturation.

The effect size of delta frequency PSDs was significantly stronger in both C3A2 and C4A1 channels than that of either alpha [mean overall difference C3A2 0.824 (95% CI 0.822, 0.827)) or beta (mean overall difference C3A2 0.827 (95% CI 0.824, 0.830)] (Table 4). The effect size of alpha PSD was significantly larger than that of beta overall and in all stages. Furthermore, within the C3A2 channel, for N3 and REM sleep the difference in effect size between alpha and beta was significantly greater than for N1 + N2 stages – however in the C4A1 channel the effect size difference was significantly smaller in N3 compared to N1 + N2 and REM. As well as significant differences in effect sizes between frequencies, there were significant differences in effect sizes between channels within all frequency bands. The effect size within the C4A1 channel was significantly smaller than the C3A2 for each frequency. For the beta frequency particularly, the effect size difference between channels was anywhere from two to five-fold greater than for the alpha and delta frequency bands.

**Table 4:** Mean differences in effect size within multivariate binomial regression between alpha, beta and delta frequencies for channels C3A2 and C4A1, and between channels for each of alpha, beta, and delta.

Wilcoxon effect size difference (95% CI)				
Channel	Sleep Stages	Delta vs. Alpha	Alpha vs. Beta	Delta vs. Beta
C3A2	N1 + N2	0.859 (0.855–0.863)	0.031 (0.027–0.035)	0.847 (0.84–0.851)
	N3	0.865 (0.859–0.872)	0.374 (0.367–0.380)	0.865 (0.858–0.871)
	REM	0.859 (0.853–0.866)	0.380 (0.373–0.387)	0.836 (0.828–0.843)
	All sleep stages	0.824 (0.822–0.827)	0.033 (0.030–0.036)	0.827 (0.824–0.830)
C4A1	N1 + N2	0.861 (0.857–0.865)	0.232 (0.228–0.236)	0.795 (0.791–0.799)
	N3	0.865 (0.858–0.872)	0.054 (0.047–0.060)	0.821 (0.814–0.827)
	REM	0.858 (0.852–0.865)	0.496 (0.490–0.504)	0.767 (0.761–0.774)
	All sleep stages	0.830 (0.827–0.833)	0.222 (0.219–0.225)	0.774 (0.771–0.777)
C3A2, C4A1	All sleep stages	0.827 (0.825, 0.829)	0.130 (0.128–0.132)	0.801 (0.799–0.803)
		Delta	Alpha	Beta
	N1 + N2	0.108 (0.104–0.113)	0.038 (0.034–0.043)	0.193 (0.188–0.197)
	N3	0.141 (0.135–0.147)	0.124 (0.118–0.131)	0.295 (0.288–0.301)
	REM	0.039 (0.032–0.046)	0.111 (0.104–0.117)	0.256 (0.249–0.263)
	All sleep stages	0.081 (0.078–0.084)	0.051 (0.048–0.054)	0.198 (0.195–0.201)
	All sleep stages	0.043 (0.041–0.044)		

CI, Confidence interval; N1/N2/N3; non-rapid eye movement stage 1/2/3; REM, rapid eye movement.

## Discussion

In this study, among patients with mild OSA, patients with a MSL < 10 min showed significantly higher relative PSDs in the delta frequency band and significantly lower PSDs in the alpha and beta bands compared to less sleepy patients. These differences in relative PSDs were consistent across sleep stages, with noted hemispherical differences. In multivariate models’ PSDs remained significant, independent predictors for EDS. However, the effect size associated with the delta frequency band was significantly greater than that of either the alpha or beta frequency bands. We chose to analyse patients with mild OSA, as EDS remains common among these patients despite a lower presence of hypoxia and sleep fragmentation, and therefore other models must be developed to understand and explain the underlying mechanics of EDS (26). Furthermore, previous studies have identified differences in polysomnographic variables among patients with mild OSA between those subjectively assessed as sleepy and non-sleepy (27, 28).

Delta waves are strongly associated with the intensity of sleep and are known to appear with greater power following periods of sleep deprivation, such that they are considered a marker of sleep drive (34, 42–44). As such it is plausible that excessive daytime sleepiness leads to stronger delta wave activity during the night as opposed to causality in the other direction. A previous study identified similar results, among patients with chronic fatigue syndrome, who were shown to have significantly increased relative delta power (45). Although the patients in the previous study were not ‘sleepy’ as those in the current study but rather ‘fatigued’, there is some overlap between fatigue and sleepiness. Morisson et al. (46) and Xirometris et al. (47) also reported significantly greater relative delta power among patients with OSA compared to controls, with Xirometris et al. further reporting a significant positive correlation between relative delta power and ESS score. However, some key differences are noted in the current study – neither of these previous studies found a significant association between OSA/sleepiness and delta power in the central region specifically, whereas our study did so. Furthermore, our study also found significant differences in alpha and beta powers, whereas the previous studies did not. This may be due to differences in patient selection, with the current study recruiting patients with OSA and complaints of daytime sleepiness and comparing between those with an MSL <10 min, and those with an MSL ≥10 min, while the previous studies compared patients with OSA to controls without OSA. Furthermore, all patients in this study were referred on the basis of self-reported sleepiness, and as such our “non-sleepy” patients can only be considered so in this particular population context and are not relatable to “non-sleepy” individuals in the general population, nor to those perhaps in other studies. This may also be underlying the very small odds ratios noted for all factors other than sex in the multivariate models.

It has been reported that absolute power increases across all frequencies in response to apnoeic events (48), thus one would expect to see differences in absolute band powers between patients with a greater AHI and those with a lower AHI, but differences in relative band powers may not be visible. In the current study however, there was no significant difference in the AHI between sleepy and non-sleepy groups. Additionally, there are inconsistencies within the literature regarding the thresholds to be used for frequency analyses which may vary by up to 2 Hz in either direction from AASM stated thresholds of 4 Hz, 8 Hz & 13 Hz (32, 47, 49–51), which may in part explain differences in significance and effect size of findings.

Hemispheric coherence has previously been reported to be high in EDS patients (52). Yet, in the current study we noted significant differences between the C4A1 & C3A2 channels in both sleepy and non-sleepy patients, and in each of N1 + N2, N3 and REM sleep stages. Delta frequency relative power was lower in C4A1 compared to C3A2, whereas alpha and beta frequency powers were higher in C4A1 compared to C3A2. It has been reported that during sleep onset and at lower levels of arousal the right hemisphere is dominant (53), yet we noted increased delta activity and reduced alpha and beta in the right hemisphere. Furthermore, we noted statistically significant differences in the effect size for predicting EDS between C4A1 and C3A2 channels in each of N1 + N2 & N3, for all frequency bands (Table 4). The differences were relatively small, with a combined sleep stage difference of 0.081 (0.078, 0.084) in the delta frequency and 0.051 (0.048, 0.054) in the alpha frequency. However, the difference in the beta frequency was significantly larger, at 0.198 (0.195, 0.201). Further research is needed to define if hemispheric coherence is an important aspect of EDS.

Differing brain wave patterns have been noted between patients with insomnia, narcolepsy and a variety of other psychiatric disorders in comparison to controls. A greater beta power density has been noted during NREM among insomnia patients compared to healthy sleepers (35, 54), while patients with narcolepsy show higher alpha power in REM than controls (36). Seemingly in contrast, the results from the current study show increasing alpha and beta power, regardless of sleep stage, are associated with significantly reduced odds of EDS – highlighting a potential difference in the way these disorders manifest on the EEG. This may be due to the use of relative as opposed to absolute PSDs within which the heightened delta power obscures the ‘true power’ of the other frequency bands. However, as alpha power is associated with relaxed wakefulness, and beta power with active wakefulness it stands to reason that these frequencies would be lower among ‘sleepy’ patients. Additionally, among patients with sleep disordered breathing, symptoms of both depression and paranoid ideation have been associated with greater absolute power of slow oscillations (defined in the cited study as 0.5–1 Hz) (55). Previous research has shown a significant association between depression and EDS (15), which may contribute to why in the current study, increasing delta power was associated with increased odds for EDS.

Another novel method of assessing the correlation between EEG signals and EDS is the odds ratio product (ORP) (56, 57). The ORP differs from PSDs in several key ways. First, in PSDs delta frequency range typically used is 0.5-4 Hz, whereas in the ORP the thresholds used for the lower frequencies are 0.33–2.33 Hz and 2.33–6.7 Hz. Secondly the ORP is calculated in 3-s intervals compared to the 30 s epochs of the PSDs. Finally, the ORP is defined against an external reference standard (56 clinical PSGs including patients with a range of sleep disorders) while relative PSDs are normalised within each patient (56). The ORP is overall a more complex measure, showing the relationship of the powers of different EEG frequencies within a single index, while in comparison PSDs show the power of a single frequency range. Given that there are large interindividual differences in power spectra, the relative PSDs will also differ significantly based on the profile of the population under study (58). As yet however, although the ORP has shown to be significantly associated with ESS scores, no studies have utilised it to compare to following day MSLTs (59). Overall, given the novelty of the ORP, there is little literature testing the association between it and measures of sleepiness.

## Limitations

Patients recruited into this study self-reported subjective sleepiness, and thus, although they were divided into two groups based on objectively assessed MSL, there is a significant difference between the ‘non-sleepy controls’ in this study (who showed a median MSL of 14.5 min), and what may be considered ‘non-sleepy/healthy controls’ in the general population. Furthermore, we utilised a 10 min MSL cut-off for sleepy/non-sleepy groups, which differs from the 8 min cut-off used in the AASM criteria for narcolepsy, nor did we consider any REM periods during the MSLT. This study excluded patients with moderate or severe OSA, which counted for 82.5% of the patient sample with demographic information, acceptable EEGs and successful MSLTs, and therefore introduced selection bias – thus these results apply only to mild OSA and cannot be generalised to OSA more broadly. The utilised dataset also lacked clinical comorbidity and medication data, which would have significant impacts on EDS and/or EEG activity. Furthermore, we did not have information on whether patients smoked, consumed caffeine, or drank alcohol prior to the sleep study, nor did we have available any measure of subjective sleepiness such as the ESS. Additionally, as was explored above, relative powers were used in the current study, and this may limit generalisability and comparison to other studies which used absolute powers, or other power transformations. Finally, the overnight polysomnography occurred on only the single occasion, and thus may be limited by the first night effect and the patients state of sleep deprivation prior to the sleep study.

## Conclusion

These results show that there are significant differences in PSDs between sleepy and non-sleepy patients with mild OSA as measured objectively via MSLT. Sleepy patients with mild OSA show significantly greater intensity of slow waves during the night, and correspondingly lesser intensity of fast waves, even after accounting for sleep stages, and other polysomnographic and demographic parameters. Furthermore, there appear to be hemispherical differences in frequency band powers among patients with EDS compared to less sleepy patients. Further research is needed to corroborate our findings, and to assess both the impact of a greater severity of OSA and the influence of potential confounders such as cardiorespiratory comorbidities on these results.

## Data availability statement

The data analysed in this study is subject to the following licenses/restrictions: This manuscript utilises proprietary data. Requests to access these datasets should be directed to Loewenstein Hospital Rehabilitation Centre.

## Ethics statement

The studies involving humans were approved by Ethical Committee of the Loewenstein Hospital – Rehabilitation Center (0006-17-LOE). The studies were conducted in accordance with the local legislation and institutional requirements. Written informed consent for participation was not required from the participants or the participants' legal guardians/next of kin in accordance with the national legislation and institutional requirements.

## Author contributions

TH: Methodology, Supervision, Validation, Writing – original draft, Writing – review & editing. MT: Methodology, Formal analysis, Writing – original draft. Writing – review & editing. TK: Methodology, Software, Supervision, Writing – review & editing, Investigation, Validation, Writing – original draft. MR: Writing – review & editing, Supervision. HP: Writing – review & editing. AO: Writing – review & editing, Data curation, Resources. SN: Conceptualization, Methodology, Project administration, Supervision, Validation, Writing – review & editing.
