# Supplementary material for: Excessive daytime sleepiness is associated with relative delta frequency power among patients with mild OSA
Source: Front Neurol. 2024 Apr 5;15:1367860. doi: 10.3389/fneur.2024.1367860 (PMC11026663; doi:10.3389/fneur.2024.1367860)
Supplement: Supplementary file 1 [file Table_1.DOCX]

**Supplement 1.** Median and interquartile range (IQR) of the relative electroencephalogram power spectral densities (PSD) between sleepy and non-sleepy groups.

| **Channel** | **Fr** | **Groups** | **N1+N2** | | **N3** | | **REM** | |
| --- | --- | --- | --- | --- | --- | --- | --- | --- |
|  |  |  | **Median (IQR**) | ***p*-value** | **Median (IQR**) | ***p*-value** | **Median (IQR**) | ***p*-value** |
| **C3A2** | **Delta** | **Non-sleepy** | 0.347 (0.269-0.417) | <0.01 | 0.378 (0.303-0.444) | <0.01 | 0.295 (0.233-0.354) | <0.01 |
|  |  | **Sleepy** | 0.362 (0.287-0.434) |  | 0.380 (0.312-0.447) |  | 0.318 (0.287-0.434) |  |
|  | **Alpha** | **Non-sleepy** | 0.070 (0.042-0.100) | <0.01 | 0.025 (0.014-0.044) | 0.041 | 0.083 (0.054-0.113) | <0.01 |
|  |  | **Sleepy** | 0.064 (0.041-0.098) |  | 0.026 (0.017-0.041) |  | 0.079 (0.041-0.098) |  |
|  | **Beta** | **Non-sleepy** | 0.065 (0.035-0.108) | <0.01 | 0.018 (0.010-0.033) | <0.01 | 0.101 (0.063-0.139) | 0.192 |
|  |  | **Sleepy** | 0.065 (0.039-0.103) |  | 0.020 (0.012-0.033) |  | 0.099 (0.039-0.103) |  |
| **C4A1** | **Delta** | **Non-sleepy** | 0.335 (0.249-0.403) | <0.01 | 0.362 (0.280-0.427) | <0.01 | 0.291 (0.228-0.350) | <0.01 |
|  |  | **Sleepy** | 0.351(0.272-0.420) |  | 0.365 (0.294-0.431) |  | 0.315 (0.272-0.424) |  |
|  | **Alpha** | **Non-sleepy** | 0.071 (0.044-0.103) | <0.01 | 0.031 (0.017-0.055) | <0.01 | 0.089 (0.061-0.121) | <0.01 |
|  |  | **Sleepy** | 0.066 (0.044-0.099) |  | 0.028 (0.018-0.044) |  | 0.081 (0.044-0.099) |  |
|  | **Beta** | **Non-sleepy** | 0.081 (0.039-0.132) | <0.01 | 0.025 (0.012-0.057) | <0.01 | 0.111 (0.069-0.164) | <0.01 |
|  |  | **Sleepy** | 0.072 (0.043-0.114) |  | 0.023 (0.014-0.040) |  | 0.109 (0.043-0.114) |  |
